# Differences in work injury risk between immigrants and natives: changes since the economic recession in Italy

**DOI:** 10.1186/s12889-019-7178-2

**Published:** 2019-06-27

**Authors:** Massimiliano Giraudo, Antonella Bena, Michele Mosca, Elena Farina, Roberto Leombruni, Giuseppe Costa

**Affiliations:** 1 Department of Epidemiology, ASL TO3, via Sabaudia 164, 10095 Grugliasco, Italy; 2 Department of Economics Cognetti de Martiis, University of Turin, Lungo Dora Siena, 100 A, 10153 Turin, Italy; 3 Department of Clinical and Biological Sciences, University of Turin, Regione Gonzole 10, 10043 Orbassano, Italy

**Keywords:** Immigrant workers, Occupational injuries, Economic crisis, Work and health histories Italian panel, Longitudinal study

## Abstract

**Background:**

It is known that occupational injury rates are higher for immigrant than for native workers, however the effects of the economic cycles on these differences has not been assessed to date. The aim of the paper is to test if the crisis has the same mechanism of selection in the two groups by comparing injury rates in 2005 (before the crisis) and in 2010 (after the crisis).

**Methods:**

The Work History Italian Panel-Salute integrated database was interrogated to identify employment contracts in the metalworking and construction industries for the years 2005 and 2010 and the occupational injuries. A definition based on the type of injury, less likely to be biased by underreporting, was used to select serious events. Immigrants and natives were matched using the propensity score method and injury rates were calculated in the two years. Analyses were stratified by industry.

**Results:**

In the metalworking industry injury rates slightly increased over time for both groups, and were higher among immigrant than native workers in both 2005 and 2010. In the construction industry the 2005 injury rate was the same in the two groups, and there was a negative trend over time in both groups. However the decline in the 2010 injury rate for Italian workers was much larger, which led to a considerable increase of the incidence rate ratio of immigrants with respect to native (IRR 3.83, 95% CI 2.52–5.75).

**Conclusions:**

The economic recession had an impact on the risk of workplace injury. Though the main observed factors (18 variables) usually reported in literature to explain the higher injury rates of the immigrant workers were controlled through the matching, there were still differences between immigrants and natives. The main reason is that immigrants continue to be assigned to the more dangerous jobs and the more dangerous tasks within these job. Furthermore, also differences in the perception of workplace injury risks, linguistic barriers, and cultural factors may have a role in explaining this gap.

**Electronic supplementary material:**

The online version of this article (10.1186/s12889-019-7178-2) contains supplementary material, which is available to authorized users.

## Background

The risk of total and fatal occupational injury is generally higher for immigrant than for native workers, although some study results are discordant [1, 2]. The higher injury rates among immigrant workers in Italy differ in the relative risk depending on worker nationality and economic sector [3]. The reasons mainly regard the assignment of immigrant workers to the more dangerous jobs and the more dangerous tasks within these jobs, and the transient nature of their employment situation (immigrant workers transition between unemployment, underemployment, and informal labour force participation) [4]. Furthermore, immigrant workers are more often employed by small firms where the risk of fatal and serious workplace accidents is higher than in large companies [5]. Other factors are the differences in the perception of work-related risks, linguistic barriers, and cultural factors that reduce the effectiveness of training [6]. As in many highly developed countries, [7] so too in Italy immigrant workers are more frequently employed under fixed-term contracts [8]. 

Since 2008 Italy has been caught in an economic recession. The unemployment rate rose from 6.1% in 2007 to 8.4% in 2010, while the rate among immigrant workers increased from 8.3% in 2007 to 11.6% in 2010 [9]. The employment status of immigrants has worsened, with a greater increase in the share of underemployed immigrant workers [10]. Despite the recession, the number of residence permits issued for labour reasons (which are the migrants’ entry channel) continued to grow until 2010 [11, 12]. This was due in part to the entry of Eastern European countries in the European Union in 2008, in part to a persisting, although controversial, need for foreign manpower, which has convinced decision-makers to maintain legal channels relatively open also in times of crisis. Since 2008 Romanians have represented the largest proportion of immigrants, followed by Albanians and Moroccans.

There is a procyclical relationship between economic growth and occupational injuries in the short term. The injury rate rises during periods of economic growth and falls during recessions [13–15]. Also a recent study has shown that in Italy workplace injury rates in manufacturing declined between 1994 and 2012, and this downward trend was further accelerated after 2008 [16]. The main assumptions underlying this association are changes in workforce composition, working conditions, reporting behaviour and mix of employment sectors. Furthermore de la Fuente suggests that the economic recession seems to exert a sort of “natural selection” in the labour market where only the most fit tend to remain employed, with a far lower probability of sustaining a workplace injury [17].

To the best of our knowledge, no studies to date have assessed how the current recession is affecting the work safety of immigrant workers. We know that they start from a disadvantaged condition, but our hypothesis is that the economic crisis has the same mechanisms of selection among immigrants and among natives so the differential in injury rates between the two groups should disappear after the economic crisis. To study this hypothesis we compared work injury rates of immigrant and native workers in 2005 (two years prior to the start of the recession) and in 2010, controlling both from confounders and mediators usually considered in the literature to explain injury risk differences, in order to assess an eventual net impact of the crisis over and above these factors.

## Materials and methods

### Definition of immigrant

To define immigrants a criteria based on the country of birth was used, and two groups were formed: people born in high income countries (HIC, as defined by the World Bank; Italians made up 98% of this group) and people born in countries with strong migratory pressure (SMPC: Africa; Middle East and Asia excluding Israel, South Korea, and Japan; Latin America; Central and Eastern Europe). This definition of immigration status was developed within a specific project, financed by the Italian Ministry of Health, which had the aim of studying the health of immigrant population [18]. The immigrant population does not therefore correspond to the foreign one. This distinction is useful to detect the subjects that come from more deprived countries, who usually tend to accept worse working conditions.

### Data sources

The Work Histories Italian Panel (WHIP) database contains individual work histories developed from the administrative archives of the National Institute for Social Welfare (INPS). It was built starting from a systematic sample of workers selected every year on the basis of the day of birth (1st and 9th of each month), and it represents the 7% of the reference population. A career path was reconstructed for each person considering work periods, retirement and unemployment benefits. Currently, the historical series covers the period 1985 to 2012.

The WHIP sample is representative of the workers registered at the INPS, therefore it represents the private sector (manufacturing, construction and services) and does not cover public employment and agriculture. It comprises employees, self-employed and professionals, apart from some specific categories, eg. architects and lawyers, which are not registered at the INPS. The most extensive and complete data regards employees, for whom in addition to demographic characteristics, various information on jobs and companies are available.

Using the same sampling criteria (day of birth 1st and 9th of each month), occupational injury claims for absence from work for more than 3 days certified by a physician (mandatory) between 1994 and 2012 were extracted from the archive of the National Insurance Institute for Occupational Injuries (INAIL). Similarly, hospital discharges between 2001 and 2014 were extracted from the archive of the Italian Ministry of Health.

These three archives were then linked via an encrypted unique identifier based on the worker’s tax code and the integrated database is called WHIP-Salute. For a more detailed description of the WHIP-Salute database, see Bena et al [19].

The hypothesis underlying the choice of using a sampling frame based on the birth date is that the probability of extracting an individual will be uniform within a given year. However this assumption is not always valid for immigrant workers because they often do not know their exact date of birth and give January 1 on registration in official records in Italy. This generates an oversampling differential by immigrant status that could create bias. To correct this distortion, we assigned a weight to each worker based on his country of birth and using the distribution of the resident population according to the 2011 Italian General Census of Population and Housing [20].

This study doesn’t entail an ethical approval. All activities, regardless of their complexity or depth, were conducted in accordance with Italian regulations on privacy (D.Lgs. 101/2018) and with the approval of the national institutes involved. From 2013, the WHIP-Salute database has been included, under the responsibility of the Ministry of Health, in the National Statistics Program that establishes what are the statistical surveys of public interest.

### Cohorts of workers

Starting from the WHIP-Salute database two cohorts of workers were considered, in order to compare a period before and a period after the start of the economic recession. All employment contracts in the metalworking and construction industries held by men, aged between 16 and 55 years, in blue collar jobs or apprentices in 2005 (year before the economic recession) or 2010 (year of the economic recession) were then selected. Permanent, fixed-term contracts, seasonal work, and on-the-job training contracts were all included. On the contrary people that appeared for the first time in the Whip-Salute dataset in either 2005 or 2010, were excluded, due to the lack of information on previous career. All restrictions have been made to limit the analysis to the categories where the presence of immigrants is relevant, eg. metalworking and construction industries are the two sectors with the largest foreign worker component (47% of the total).

### Propensity score matching

**Fig. 1:**
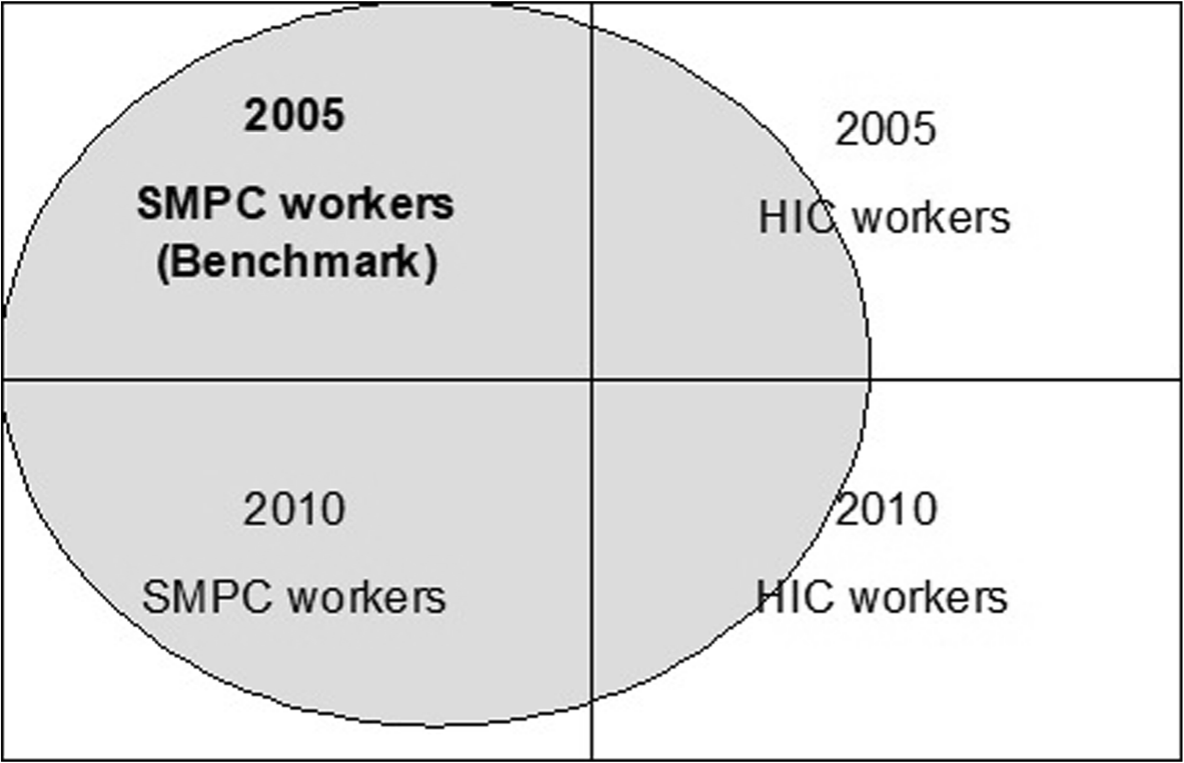
Analytical framework

The intersection between the immigration status and the year of the cohort defines four groups: SMPC workers in 2005 and 2010, HIC workers in 2005 and 2010. To make the four groups comparable a propensity score matching (PSM) was used [21]. This method allows to balance the observable covariates of exposed and unexposed groups. Matching was performed using the “PSMatching” SAS macro [22]. The analytical framework is presented in Fig. 1.

The PSM method was applied in three progressive steps with the specific aim of making all groups similar to the SMPC 2005 (benchmark group). In the first step, SMPC 2005 and HIC 2005 were considered. A logistic model was used to model the probability of being an immigrant worker in 2005, which is the propensity score (PS), Based on the PS value, each HIC worker was matched with an SMPC worker using the Kernel approach [23]. By the same procedure, in the second and third steps, the SMPC workers in 2005 were matched with the SMPC and the HIC workers in 2010. The result of these three steps was a dataset in which the four groups were observationally equal, except for their immigration status and year in which they are observed.

The variables included in all the logistic models to calculate the PS are the main factors available in WHIP-Salute which are known to be either possible confounders or mediating factors in the relation between nativity of the worker and injury risk, and which all resulted unbalanced between HIC and SMPC:

- Personal characteristic: only age was included as a continuous variable;

- Variables that describe the employment conditions: skill level (apprentice; blue collar), firm size (yearly average number of employees), geographic location of the firm (according to the country’s four administrative areas: Northwest, Northeast, Centre, South, and Islands), month in which the individual entered the cohort, and job tenure (time elapsed from the beginning of the contract and the beginning of the follow-up, continuous variable);

- Variables that describe the work career, calculated considering the 20 years preceding the beginning of the follow-up (2005 or 2010 respectively): prevailing skill level had as employee; having worked as self-employed, artisan or trader; having worked as an employee, self-employed or professional; cumulative duration of periods of employment; cumulative duration of periods of unemployment; prevailing economic sector in which the worker had worked (metalworking, construction, wholesale and retail trade, transport and storage, financial and real estate, hotel and restaurant, other manufacturing sectors, education and health services, missing data); prevailing firm size where the worker had worked; the prevailing geographic location of the firm where the worker had worked; quartile of wage (which was calculated for the 5 years preceding the beginning of the follow-up).

- Variables that describe health status considering the period preceding the beginning of the follow-up (2005 or 2010 respectively): proportion of weeks of sickness absence and of paid weeks in the 5 years preceding the beginning of the follow-up; number of serious workplace injuries in the 5 years preceding the beginning of the follow-up; number of hospital discharges in the 3 years preceding the beginning of the follow-up.

### Statistical analysis

The outcome of interest was the injury rate. As numerator, all serious workplace injuries recognized by INAIL were selected. For the present analysis an injury was defined serious if: the type of injury was an anatomic loss, a fracture, a foreign body in an eye; or the anatomic part involved was hand, wrist, arms, chest, spinal column, pelvis, hip, knee, ankle, foot; or the event was fatal. Time-at-risk was calculated on the basis of months actually worked, after subtracting all periods of absence from work due to illness or injury and temporary lay-off from paid months.

Injury rates per 1000 person years were calculated. Confidence intervals (CI) were calculated using the bootstrapping method [24]. In brief, the basic idea of bootstrapping is that inference about a population from sample data can be modelled by resampling the sample data and performing inference about a sample from the resampled data. Incidence rate ratios (IRR) were calculated to compare injury rates. The confidence interval of IRR was calculated with Byar’s approximation [25]. All analyses were stratified by industry. Statistical analyses were performed using SAS 9.3 (SAS Institute, Carey, NC, USA).

## Results

A total of 181,186 workers were observed (40,735 SMPC and 140,451 HIC). Tables 1 and 2 show the distribution of the main characteristics of the workers by industrial sector before the matching (a complete description is given in the Additional file 1 Material). In both sectors, SMPC workers were younger with respect to HIC (t-test, *p* < .0001). Moreover they were generally employed by small and medium enterprises located mainly in northern Italy (Pearson’s chi-squared test, p < .0001) and had a shorter job tenure (t-test, *p* < .0001).

**Table 1:** – Main personal, employment, work career characteristics, and health status of workers by immigration status and year of work in the metalworking industry before propensity score matching

		2005				2010			
		HIC^a^		SMPC^b^		HIC^a^		SMPC^b^	
		Person years	%	Person years	%	Person years	%	Person years	%
		63,605	100	7505	100	53,984	100	8487	100
Worker characteristics									
Age	< 25	7584	11.9	796	10.6	4608	8.5	793	9.3
	25–34	21,474	33.8	2742	36.5	14,859	27.5	2554	30.1
	35–44	19,729	31.0	2777	37.0	19,040	35.3	3181	37.5
	45–55	14,817	23.3	1189	15.8	15,477	28.7	1958	23.1
Employment									
Job tenure (in months)	< 12 months	12,049	18.9	2586	34.5	7390	13.7	1967	23.2
	1–4 years	18,887	29.7	3557	47.4	14,916	27.6	3585	42.2
	5–9 years	13,499	21.2	1010	13.5	11,910	22.1	2138	25.2
	10–14 years	7171	11.3	243	3.2	8477	15.7	595	7.0
	≥ 15 years	11,998	18.9	110	1.5	11,291	20.9	201	2.4
Firm size (yearly average no. of employees)	1–9	12,515	19.7	1996	26.6	10,921	20.2	2160	25.5
	10–19	9282	14.6	1561	20.8	7849	14.5	1712	20.2
	20–199	23,569	37.1	3024	40.3	20,096	37.2	3600	42.4
	> 199	18,239	28.7	924	12.3	15,118	28.0	1015	12.0
Firm’s geographic location	Northwest	24,681	38.8	3111	41.5	20,131	37.3	3472	40.9
	Northeast	18,174	28.6	3198	42.6	15,757	29.2	3516	41.4
	Central	8483	13.3	907	12.1	7493	13.9	1047	12.3
	South and Islands	12,267	19.3	288	3.8	10,604	19.6	452	5.3
Working career (in the 20 years preceding the year)									
Prevailing skill level	Apprentice	5886	9.3	516	6.9	4032	7.5	588	6.9
	Blue collar	56,936	89.5	6976	93.0	49,195	91.1	7876	92.8
	White collar	782	1.2	12	0.2	756	1.4	22	0.3
Cumulative duration as employee	< 12 months	1502	2.36	421	5.62	671	1.2	248	2.9
	1–4 years	8863	13.9	3422	45.6	5736	10.6	2442	28.8
	5–9 years	12,581	19.8	2123	28.3	8910	16.5	3202	37.7
	10–14 years	16,164	25.4	1005	13.4	15,559	28.8	1747	20.6
	≥ 15 years	24,495	38.5	533	7.11	23,109	42.8	848	10.0
Quartile of wage in the 5 years preceding the beginning of follow-up	I	12,601	19.8	2765	36.8	10,083	18.7	3121	36.8
	II	14,305	22.5	2551	34	12,001	22.2	2693	31.7
	III	17,285	27.2	1498	20	14,732	27.3	1753	20.7
	IV	19,415	30.5	691	9.21	17,169	31.8	920	10.8
Prevailing economic sector	Metalworking	53,983	84.9	5811	77.4	46,801	86.7	7072	83.3
	Construction	2329	3.66	406	5.41	1970	3.6	442	5.2
	Wholesale and retail trade	1743	2.74	109	1.46	1196	2.2	92	1.1
	Transport, storage, and communication	417	0.65	100	1.33	313	0.6	55	0.6
	Financial and real estate	998	1.57	465	6.2	926	1.7	425	5.0
	Hotel and restaurant	339	0.53	100	1.33	217	0.4	66	0.8
	Other manufacturing	3170	4.98	411	5.47	2113	3.9	271	3.2
	Instruction and health services	200	0.31	28	0.38	162	0.3	22	0.3
	Missing	428	0.67	75	0.99	285	0.5	42	0.5
Health status in the 5 years preceding the beginning of follow-up									
Ratio between no. of weeks of sickness absence and total paid weeks (in %) in the 5 years preceding the beginning of follow-up	Missing	123	0.19	7	0.1	109	0.2	18	0.2
	0	34,006	53.5	4474	59.6	29,477	54.6	5216	61.5
	1–4	21,352	33.6	2182	29.1	18,232	33.8	2539	29.9
	5–9	5788	9.1	566	7.5	4486	8.3	520	6.1
	10–19	1975	3.1	231	3.1	1469	2.7	163	1.9
	20–49	344	0.54	37	0.5	202	0.4	30	0.4
	50–74	11	0.02	4	0.1	6	0.0	2	0.0
	75–100	6	0.01	1	0.0	4	0.0	0	0.0

a: HIC - high income country b: SMPC - strong migratory pressure country

**Table 2:** Main personal, employment, work career characteristics, and health status of workers by immigration status and year of work in the construction industry before propensity score matching

		2005				2010			
		HIC^a^		SMPC^b^		HIC^a^		SMPC^b^	
		Person years	%	Person years	%	Person years	%	Person years	%
		45,661	100	11,182	100	40,142	100	12,857	100
Worker characteristics									
Age	< 25	8064	17.7	1710	15.3	5297	13.2	1749	13.6
	25–34	13,672	29.9	4658	41.7	11,103	27.7	4990	38.8
	35–44	13,363	29.3	3453	30.9	12,324	30.7	4232	32.9
	45–55	10,562	23.1	1362	12.2	11,418	28.4	1887	14.7
Employment									
Job tenure (in months)	< 12 months	20,095	44.0	6527	15.3	15,270	38.0	5966	46.4
	1–4 years	15,922	34.9	4102	41.7	13,813	34.4	5184	40.3
	5–9 years	5456	11.9	449	30.9	6176	15.4	1445	11.2
	10–14 years	2011	4.4	94	12.2	2511	6.3	207	1.6
	≥ 15 years	2176	4.8	11	0.1	2373	5.9	56	0.4
Firm size (yearly average no. of employees)	1–9	24,249	53.1	7042	63.0	21,803	54.3	7609	59.2
	10–19	8706	19.1	1797	16.1	7437	18.5	2312	18.0
	20–199	10,643	23.3	2171	19.4	9247	23.0	2788	21.7
	> 199	2063	4.5	172	1.5	1655	4.1	148	1.2
Firm’s geographic location	Northwest	11,852	26.0	4423	39.6	9881	24.6	4885	38.0
	Northeast	9100	19.9	3194	28.6	7416	18.5	3091	24.0
	Central	8191	17.9	2719	24.3	7108	17.7	3601	28.0
	South and Islands	16,518	36.2	846	7.6	15,736	39.2	1279	10.0
Working career (in the 20 years preceding the year)									
Prevailing skill level	Apprentice	7522	16.5	991	8.9	5266	13.1	1683	13.1
	Blue collar	37,730	82.6	10,183	91.1	34,484	85.9	11,167	86.9
	White collar	409	0.9	7	0.1	392	1.0	7	0.1
Cumulative duration as employee	< 12 months	2443	5.4	1039	9.3	1334	3.3	944	7.3
	1–4 years	10,283	22.5	6485	58	7576	18.9	5182	40.3
	5–9 years	10,714	23.5	2512	22.5	9684	24.1	4280	33.3
	10–14 years	10,655	23.3	731	6.54	10,108	25.2	1425	11.1
	≥ 15 years	11,565	25.3	415	3.71	11,439	28.5	1026	8.0
Quartile of wage in the 5 years preceding the beginning of follow-up	I	10,918	23.9	2609	23.3	9070	22.6	3164	24.6
	II	9174	20.1	3531	31.6	8316	20.7	3657	28.4
	III	10,953	24	3154	28.2	9461	23.6	3667	28.5
	IV	14,615	32	1888	16.9	13,295	33.1	2370	18.4
Prevailing economic sector	Metalworking	2692	5.9	450	4.02	1792	4.5	312	2.4
	Construction	37,376	81.9	9446	84.5	34,622	86.3	11,845	92.1
	Wholesale and retail trade	1137	2.49	169	1.51	785	2.0	89	0.7
	Transport, storage, and communication	559	1.22	118	1.06	389	1.0	72	0.6
	Financial and real estate	592	1.3	292	2.61	494	1.2	181	1.4
	Hotel and restaurant	422	0.92	152	1.36	310	0.8	79	0.6
	Other manufacturing	2042	4.47	394	3.53	1330	3.3	199	1.6
	Instruction and health services	338	0.74	32	0.29	206	0.5	21	0.2
	Missing	502	1.1	128	1.15	214	0.5	58	0.5
Health status in the 5 years preceding the beginning of follow-up									
Ratio between no. of weeks of sickness absence and total paid weeks (in %) in the 5 years preceding the beginning of follow-up	Missing	401	0.88	24	0.2	340	0.8	73	0.6
	0	26,085	57.1	7959	71.2	23,018	57.3	9264	72.0
	1–4	12,627	27.7	2074	18.5	11,506	28.7	2570	20.0
	5–9	4019	8.8	683	6.1	3460	8.6	629	4.9
	10–19	1947	4.26	322	2.9	1425	3.5	244	1.9
	20–49	527	1.15	105	0.9	375	0.9	71	0.6
	50–74	42	0.09	12	0.1	13	0.0	3	0.0
	75–100	13	0.03	3	0.0	6	0.0	4	0.0

a: HIC - high income country b: SMPC - strong migratory pressure country

The HIC workers had a longer working career than the SMPC workers and they received a higher wage (t-test, *p* < .0001). Considering only workers in the metalworking industry (Table 1) more HIC workers were employed in the metalworking sector also in the past (84.9% in 2005 and 86.7% in 2010) than SMPC workers (77.4% in 2005 and 83.3% in 2010). Considering only workers in the construction industry (Table 2) more HIC workers were employed in the construction sector also in the past (81.9% in 2005 and 86.3% in 2010) than SMPC workers (84.5% in 2005 and 92.1% in 2010). Finally, SMPC workers submitted fewer sick-leave claims than HIC workers (t-test, *p* < .0001).

The matching method allowed us to link the four groups. Evaluation of the appropriateness of the matched samples and of the success of propensity score modelling is given in the Additional file 2 Material. To summarise, the standardized differences were strongly reduced after the matching and the Sianesi-test [26] confirmed that, after matching, there were no systematic differences in the distribution of covariates between groups. The pseudo-R2 calculated on the matched sample was close to zero.

Table 3 presents the injury rates and IRR by immigration status in the metalworking sector after PSM. The workplace injury rates of the HIC workers were generally lower than those of the SMPC workers, and this difference was statistically significant. Looking at the trend over time, the 2010 rate was higher than the 2005 rate for both the SMPC and the HIC workers. The increase was not statistically significant, indicating that the difference in risk between the SMPC and the HIC workers remained unchanged over time.

**Table 3:** Injury rate per 1000 workers and Incidence Rate Ratio (IRR) (reference: SMPC 2005) in the metalworking industry after propensity score matching [IRR: incidence rate ratio; CI = confidence interval]

	HIC^a^					SMPC^b^				
	No. of injuries	Injury rate	95% CI	IRR	95% CI	No. of injuries	Injury rate	95% CI	IRR	95% CI
2005	33	4.24	(3.20–5.27)	0.37	(0.24-0.54)	86	11.49	(9.12–13.86)	1	–
2010	49	6.36	(4.90–7.81)	0.55	(0.39–0.79)	98	13.37	(10.08–16.66)	1.16	(0.87–1.55)

a: HIC - high income country b: SMPC - strong migratory pressure country

In the construction industry, the 2005 injury rate was the same for the HIC workers as for the SMPC workers (Table 4). The injury rate for the SMPC workers didn’t change over time (IRR SMPC 2010 vs. SMPC 2005 0.84; 95% confidence interval (CI) 0.65–1.09), whereas a drastic decline in the 2010 injury rate was noted for the HIC workers, with the effect that the risk difference between the SMPC and HIC workers changed significantly over time. While the 2005 injury rates were substantially identical, the IRR between the SMPC and the HIC groups was 3.83 in 2010 (95% CI 2.52–5.75).

**Table 4:** Injury rate per 1000 workers and Incidence Rate Ratio (IRR) (reference: SMPC 2005) in the construction industry after propensity score matching [IRR: incidence rate ratio; CI = confidence interval]

	HIC^a^					SMPC^b^				
	No. of injuries	Injury rate	95% CI	IRR	95% CI	No. of injuries	Injury rate	95% CI	IRR	95% CI
2005	33	13.34	(6.12–20.56)	1.19	(0.94–1.50)	125	11.24	(9.47–13.00)	1	–
2010	49	2.47	(1.85–3.10)	0.22	(0.15–0.33)	103	9.46	(6.71–12.21)	0.84	(0.65–1.09)

a: HIC - high income country b: SMPC - strong migratory pressure country

## Discussion

We studied the hypothesis that economic crisis had the same mechanisms of selection among SMPC workers and HIC workers and that, taking into account the main factors that could be associated with health and employment conditions, the differential in injury rates between the two groups would tend to disappear from before to after the recession. With this aim, we compared the injury rates between groups and over time.

To make the comparison as free as possible from potential source of bias we applied some methodological choices: we used the PSM method to control for differences between groups and variations in the workforce composition over time; we considered only serious injuries to control for the phenomenon of underreporting; and we stratified the analyses by economic sector in which immigrant are mainly represented.

The descriptive analysis underlines the marked differences between SMPC and HIC workers and the changes in the workforce composition since the start of the economic recession. These results confirm the need to use a method to control for confounding in order to compare the groups of workers. The average age of both the SMPC and the HIC groups rose between 2005 and 2010. Selection of experienced workers during the recession could be seen in the increase in the average job tenure. This finding was also present when we observed how the distribution of the cumulative duration as employee changed between 2005 and 2010. These results are consistent with the official statistics on Italian labour market [9].

The variables included in the regression model are the main factors commonly used to explain injury risk differences between SMPC and HIC workers. They represent the principal characteristics of employment conditions, an individual’s work career, and health status. Among other factors, we wish to emphasize the use of the “job tenure” variable, which is an important determinant of the risk of work-related injury. Most published research provides evidence that newly hired workers – whatever the contract type – are more likely to sustain an injury than those with a longer job tenure, even after taking into account background variables and previous experience [27, 28]. In a context where labour market flexibility is increasing and changing jobs is becoming more common, workers repeatedly find themselves working in an initially “high-risk” period. This precarious employment situation is particular to immigrant workers [29].

During the recession, the mean number of weeks of sickness absence registered by INPS decreased for the SMPC and the HIC workers in both the metalworking and the construction industries. This change can be interpreted as an increase of presenteeism in the workplace, especially among the SMPC workers [30]. To take into account this phenomenon we decided to use only a subset of serious injury which are unlikely to be underreported. In general, serious injury rates are less affected by underreporting with respect to minor injuries or total injuries [13, 31]. However, different definition of seriousness can be adopted, which can lead to different level of underreporting. The definition used in this paper to identify serious injuries avoid using time off from work, often used as a proxy of injury severity, since there were reported practices of early return to work for foreign workers or underreporting for events with a long prognosis [32].

Finally all analyses were stratified by sectors because the association and the mechanisms through which the business cycle affects the incidence of workplace injuries may differ across industries [33].

Results presented in Tables 3 and 4 provide many food for thought.

In the metalworking industry (Table 3), for both groups of workers injury rates grown a little over time, even if the difference was not statistically significant. Though we took into account the workers’ principal characteristics, the risk differentials between the SMPC and the HIC groups from before to after the crisis was the same. This indicate that PSM allowed to manage the confounding related to changes in workforce composition, but also that mechanisms of selection determined by unmeasured factors were the same in both groups. Injury rates for HIC workers remain lower than those of SMPC both before and after the recession (IRR 0.55; 95% CI 0.39–0.79). This may be explained by differences in the level of education, the perception of job-related risk, the knowledge of the Italian language and the participation in training courses. Furthermore, immigrant workers may find it more difficult to adapt themselves to the work organization [34]. Another important factor to consider is the assignment of immigrants to the more dangerous jobs and to the more dangerous tasks within these jobs [4]. Also, the possibility of negotiating between such tasks among SMPC workers is probably lower as compared to HIC workers.

No change in the workplace injury rates for SMPC workers over time was noted in the construction industry (IRR 0.84; 95% CI 0.65–1.09) after matching (Table 4). This confirms that the risk differences were nullified between the SMPC workers in 2005 and those with the same characteristics in 2010. At the same time in 2005, the risk of injury between SMPC and HIC workers was the same. The unexpected result was the drop in the construction injury rate of the HIC workers from 2005 to 2010, with a resulting rise in the risk differential between the SMPC and the HIC workers (IRR 0.22; 95% CI 0.15–0.33). Although the PSM selected workers of the HIC group with characteristics similar to the workers of the SMPC group in 2005, selection in the workforce also occurred during the economic recession. For example, some workers had to transition from contracted employment to being self-employed or entering informal work. In addition, employers tend to assign the most dangerous tasks to self-employed workers. This phenomenon involves both immigrant and native workers [35]. Our hypothesis is that there was a very strong workforce selection among the HIC workers with characteristics similar to those of SMPC workers; therefore, people still employed as employees in 2010 had the “best” profile also in terms of health, and this may have contributed to the lower injury rates. Moreover, immigrant workers, driven by economic necessity, were still more likely to accept poor working conditions while Italian workers could be more selective in their options and choices.

The strengths of this study are the main characteristics of the WHIP-Salute database: national representativeness and longitudinal nature and high quality data that allowed us to describe the characteristics of workforce participation in great detail. Most countries do not have adequate national systems that monitor key occupational health problems of immigrants, and most official and nonofficial statistics do not disaggregate migratory flows by age, gender, ethnicity, and social class.

Another strength is the use of the PSM method in the analysis. As we documented, there were large differences between HIC and SMPC workers in the exposure to many important risk factors for work injuries. To properly control for all these factors with a multiple regression model would have forced us to make strong assumptions regarding the type of association of each of them with the health outcome (linear, quadratic, etc.) and about possible interaction between them, resulting in possible miss-specification biases. The use of the PSM method was successful in creating comparison groups which were similar with respect to all those characteristics, which allowed us to interpret the residual differences we found in injury risks just to being before or after the crisis, and being HIC or SMPC workers.

The main limitation of the study regards the external validity of the results. Our research was restricted to male workers employed as blue collars or apprentices, aged between 16 and 55 years, in the metalworking or construction sector. Furthermore we excluded workers seen for the first time in the WHIP-Salute database in either the 2005 or 2010. The results for this worker category are reliable, but to verify whether our conclusions can be generalized, the analysis needs to be extended to other economic sectors not considered in this analysis, to women, and to the self-employed.

Another limitation is that the WHIP-Salute database contains information only on workers registered at the INPS. As such, it does not comprise the entire immigrant population in Italy, which also includes illegal workers and immigrants without official documents known to be at high risk of workplace accidents [32]. Furthermore, we have no information about the immigrant worker’s work career prior to his arrival in Italy. This can lead to underestimate the person’s work experience, which should have a protective effect on the risk of injury. Therefore matching a SMPC worker who seems without experience with a real new HIC worker can result in an underestimation of the IRR. This problem is partly mitigated considering the age, which is correlated with experience.

## Conclusions

Our study compares workplace injury rates between SMPC workers and HIC workers for 2005 and 2010.

Focusing on the comparisons of interest, between groups and over time, we highlight two main conclusions.

The first is that, regardless of the level in 2005, in 2010 HIC workers are protected with respect to SMPC workers in terms of injury rates, in both metalworking and construction industry. The second is that the dynamic of the impact of the economic crisis is the same in HIC and SMPC workers within the metalworking industry, whereas in the construction industry injury rates of HIC workers had a much greater decline than SMPC workers.

Since we controlled for the main observed factors (18 variables) usually reported in literature to explain the higher injury rates of the immigrant workers, the reasons for the persistence of differences have to be found elsewhere. In our opinion, the main reason is that immigrant workers are assigned to do the more dangerous jobs and the more dangerous tasks within these job. Furthermore, also differences in the perception of workplace injury risks, linguistic barriers, and cultural factors may have a role in explaining this gap.

The study of the mechanisms that explain the differential health of immigrant workers has been identified as a priority for research, and this issue is indicated as a priority for a global agenda in occupational health [36, 37]. The Whip-Salute database allowed us to analyse high quality data in great detail.

These results represent an initial step in the analysis of the effects of the current economic recession on the risk of occupational injury by comparing the condition of immigrant workers before and during the recession. A future area of focus should examine how work safety has changed for immigrant workers, considering also specific country of birth, and the transformations that immigration flows have produced.

## Additional files

Additional file 1: Characteristics of workers by immigration status and year of work before the PS matching (DOCX 34 kb)

Additional file 2: Appropriateness of the matched samples (DOCX 36 kb)

## Data Availability

The datasets generated during and/or analysed during the current study are not publicly available, but are available from the corresponding author on reasonable request. From 2013, the WHIP-Salute database has been included, under the responsibility of the Ministry of Health, in the National Statistics Program (NSP) that establishes what are the statistical surveys of public interest. Our institutions are included in the NSP form with the role of developer of the final database, so the data that we used was openly available to us. The Ministry of Health releases microdata files for research purposes, upon request based on a research protocol and after authorization of the Italian Data Protection Authority.

## Abbreviations

CI: Confidence interval
HIC: highly developed countries as defined by the World Bank
INAIL: Italian workers compensation authority
INPS: Italian National Social Security Institute
IRR: Incidence rate ratio
ISTAT: Italian National Institute of Statistics
PS: propensity score
PSM: propensity score matching
SMPC: countries with strong migratory pressure
WHIP-Salute: Work and Health Histories Italian Panel-Health
