# Supplementary material for: Differences in work injury risk between immigrants and natives: changes since the economic recession in Italy
Source: BMC Public Health. 2019 Jun 27;19:836. doi: 10.1186/s12889-019-7178-2 (PMC6598376; doi:10.1186/s12889-019-7178-2)
Supplement: Supplementary file 1 — Characteristics of workers by immigration status and year of work before the PS matching (DOCX 34 kb) [file 12889_2019_7178_MOESM1_ESM.docx]

**Additional file 1**

**Characteristics of workers by immigration status and year of work before the PS matching (variables not included in table 1 and 2 of the article).**

**Table 1 – Employment characteristics in the metal working sector.**

|  | | **2005** | | | | **2010** | | | |
| --- | --- | --- | --- | --- | --- | --- | --- | --- | --- |
|  |  | HIC^a^ | | SMPC^b^ | | HIC^a^ | | SMPC^b^ | |
|  |  | Person years | % | Person years | % | Person years | % | Person years | % |
| **Total** | | 63,605 | 100 | 7,505 | 100 | 53,984 | 100 | 8,487 | 100 |
|  | | | | | | | | | |
| Skill level | Apprentice | 4,059 | 6.4 | 412 | 5.5 | 3,239 | 6 | 479 | 5.6 |
|  | Blue-collar | 59,545 | 93.6 | 7,093 | 94.5 | 50,745 | 94 | 8,007 | 94.3 |
| Month to start the follow-up | January | 58,111 | 91.4 | 6,362 | 84.8 | 50,065 | 92.7 | 7,471 | 88 |
|  | February | 842 | 1.3 | 188 | 2.5 | 577 | 1.1 | 132 | 1.6 |
|  | March | 805 | 1.3 | 192 | 2.6 | 603 | 1.1 | 132 | 1.6 |
|  | April | 766 | 1.2 | 146 | 1.9 | 488 | 0.9 | 115 | 1.4 |
|  | May | 656 | 1 | 132 | 1.8 | 462 | 0.9 | 139 | 1.6 |
|  | June | 645 | 1 | 119 | 1.6 | 472 | 0.9 | 121 | 1.4 |
|  | July | 517 | 0.8 | 92 | 1.2 | 357 | 0.7 | 102 | 1.2 |
|  | August | 233 | 0.4 | 40 | 0.5 | 159 | 0.3 | 58 | 0.7 |
|  | September | 448 | 0.7 | 96 | 1.3 | 342 | 0.6 | 91 | 1.1 |
|  | October | 319 | 0.5 | 81 | 1.1 | 252 | 0.5 | 76 | 0.9 |
|  | November | 201 | 0.3 | 43 | 0.6 | 160 | 0.3 | 37 | 0.4 |
|  | December | 62 | 0.1 | 13 | 0.2 | 47 | 0.1 | 13 | 0.2 |

a: HIC - high income country b: SMPC - strong migratory pressure country

**Table 2 – Work career characteristics of workers in the metal working sector.**

|  | | **2005** | | | | **2010** | | | |
| --- | --- | --- | --- | --- | --- | --- | --- | --- | --- |
|  |  | HIC^a^ | | SMPC^b^ | | HIC^a^ | | SMPC^b^ | |
|  |  | Person years | % | Person years | % | Person years | % | Person years | % |
| **Total** | | 63,605 | 100 | 7,505 | 100 | 53,984 | 100 | 8,487 | 100 |
|  | | | | | | | | | |
| Ever worked as artisan | No | 60,918 | 95.8 | 7,421 | 98.9 | 51,518 | 95.4 | 8,300 | 97.8 |
|  | Yes | 2,687 | 4.2 | 84 | 1.1 | 2,466 | 4.6 | 187 | 2.2 |
| Ever worked as trader | No | 62,275 | 97.9 | 7,463 | 99.4 | 52,795 | 97.8 | 8,413 | 99.1 |
|  | Yes | 1,330 | 2.1 | 42 | 0.6 | 1,189 | 2.2 | 73 | 0.9 |
| Ever worked as co-dependent or professionals | No | 62,138 | 97.7 | 7,266 | 96.8 | 51,687 | 95.7 | 8,163 | 96.2 |
|  | Yes | 1,467 | 2.3 | 238 | 3.2 | 2,297 | 4.3 | 324 | 3.8 |
| Cumulative duration of periods of non-employment | < 12 months | 43,639 | 68.6 | 5,919 | 78.9 | 39,317 | 72.8 | 6,781 | 79.9 |
|  | 1-4 years | 16,228 | 25.5 | 1,375 | 18.3 | 12,328 | 22.8 | 1,514 | 17.8 |
|  | 5-9 years | 3,262 | 5.1 | 191 | 2.5 | 2,064 | 3.8 | 181 | 2.1 |
|  | 10-14 years | 473 | 0.7 | 19 | 0.3 | 274 | 0.5 | 11 | 0.1 |
|  | ≥ 15 years | 4 | 0 | - | 0 | 1 | 0 | - | 0 |
| Prevailing work geographic area | Northwest | 24,467 | 38.5 | 3,069 | 40.9 | 20,218 | 37.5 | 3,473 | 40.9 |
|  | Northest | 17,576 | 27.6 | 3,120 | 41.6 | 15,583 | 28.9 | 3,499 | 41.2 |
|  | Central | 8,831 | 13.9 | 960 | 12.8 | 7,452 | 13.8 | 1,078 | 12.7 |
|  | South and Islands | 12,725 | 20 | 356 | 4.7 | 10,722 | 19.9 | 432 | 5.1 |
|  | Abroad | 6 | 0 | - | 0 | 8 | 0 | 4 | 0.1 |
| Prevailing Firm size | Missing | 61 | 0.1 | 11 | 0.2 | 59 | 0.1 | 6 | 0.1 |
|  | 1-9 | 10,102 | 15.9 | 1,641 | 21.9 | 8,599 | 15.9 | 1,720 | 20.3 |
|  | 10-19 | 8,638 | 13.6 | 1,324 | 17.6 | 6,843 | 12.7 | 1,364 | 16.1 |
|  | 20 - 199 | 24,112 | 37.9 | 2,749 | 36.6 | 19,516 | 36.2 | 3,091 | 36.4 |
|  | > 199 | 20,691 | 32.5 | 1,779 | 23.7 | 18,968 | 35.1 | 2,307 | 27.2 |

a: HIC - high income country b: SMPC - strong migratory pressure country

**Table 3 – Health status of workers in the metal working sector.**

|  | | **2005** | | | | **2010** | | | |
| --- | --- | --- | --- | --- | --- | --- | --- | --- | --- |
|  |  | HIC^a^ | | SMPC^b^ | | HIC^a^ | | SMPC^b^ | |
|  |  | Person years | % | Person years | % | Person years | % | Person years | % |
| **Total** | | 63,605 | 100 | 7,505 | 100 | 53,984 | 100 | 8,487 | 100 |
|  | | | | | | | | | |
| Number of serious work injuries in the 5 years preceding the beginning of follow-up | 0 | 61,756 | 97.1 | 7,241 | 96.5 | 52,654 | 97.5 | 8,190 | 96.5 |
|  | 1 | 1,772 | 2.8 | 252 | 3.4 | 1,293 | 2.4 | 276 | 3.2 |
|  | 2 | 72 | 0.1 | 10 | 0.1 | 36 | 0.1 | 19 | 0.2 |
|  | 3+ | 4 | 0 | 2 | 0 | 1 | 0 | 2 | 0 |
| Number of hospital discharges in the 3 years preceding the beginning of follow-up | 0 | 51,771 | 81.4 | 6,586 | 87.7 | 44,406 | 82.3 | 7,550 | 89 |
|  | 1 | 8,646 | 13.6 | 705 | 9.4 | 7,122 | 13.2 | 748 | 8.8 |
|  | 2 | 2,054 | 3.2 | 146 | 1.9 | 1,711 | 3.2 | 134 | 1.6 |
|  | 3+ | 1,135 | 1.8 | 68 | 0.9 | 744 | 1.4 | 54 | 0.6 |

a: HIC - high income country b: SMPC - strong migratory pressure country

**Table 4 – Employment characteristics in the construction industry.**

|  | | **2005** | | | | **2010** | | | |
| --- | --- | --- | --- | --- | --- | --- | --- | --- | --- |
|  |  | HIC^a^ | | SMPC^b^ | | HIC^a^ | | SMPC^b^ | |
|  |  | Person years | % | Person years | % | Person years | % | Person years | % |
| **Total** | | 45,661 | 100 | 11,182 | 100 | 40,142 | 100 | 12,857 | 100 |
|  | | | | | | | | | |
| Skill level | Apprentice | 5,877 | 12.9 | 982 | 8.8 | 3,925 | 9.8 | 1,470 | 11.4 |
|  | Blue-collar | 39,784 | 87.1 | 10,200 | 91.2 | 36,216 | 90.2 | 11,388 | 88.6 |
| Month to start the follow-up | January | 35,304 | 77.3 | 7,810 | 69.8 | 31,999 | 79.7 | 9,769 | 76.0 |
|  | February | 1,400 | 3.1 | 463 | 4.1 | 1,067 | 2.7 | 357 | 2.8 |
|  | March | 1,335 | 2.9 | 464 | 4.2 | 1,112 | 2.8 | 456 | 3.5 |
|  | April | 1,530 | 3.4 | 496 | 4.4 | 1,116 | 2.8 | 431 | 3.4 |
|  | May | 1,479 | 3.2 | 455 | 4.1 | 1,081 | 2.7 | 385 | 3.0 |
|  | June | 1,289 | 2.8 | 393 | 3.5 | 919 | 2.3 | 384 | 3.0 |
|  | July | 935 | 2.0 | 270 | 2.4 | 866 | 2.2 | 278 | 2.2 |
|  | August | 368 | 0.8 | 106 | 0.9 | 297 | 0.7 | 122 | 0.9 |
|  | September | 880 | 1.9 | 317 | 2.8 | 745 | 1.9 | 310 | 2.4 |
|  | October | 637 | 1.4 | 236 | 2.1 | 516 | 1.3 | 225 | 1.8 |
|  | November | 398 | 0.9 | 130 | 1.2 | 323 | 0.8 | 110 | 0.9 |
|  | December | 106 | 0.2 | 41 | 0.4 | 100 | 0.2 | 30 | 0.2 |

a: HIC - high income country b: SMPC - strong migratory pressure country

**Table 5 – Work career characteristics of workers in the construction industry.**

|  | | **2005** | | | | **2010** | | | |
| --- | --- | --- | --- | --- | --- | --- | --- | --- | --- |
|  |  | HIC^a^ | | SMPC^b^ | | HIC^a^ | | SMPC^b^ | |
|  |  | Person years | % | Person years | % | Person years | % | Person years | % |
| **Total** | | 45,661 | 100 | 11,182 | 100 | 40,142 | 100 | 12,857 | 100 |
|  | | | | | | | | | |
| Ever worked as artisan | No | 42,027 | 92.0 | 10,901 | 97.5 | 35,954 | 89.6 | 11,960 | 93.0 |
|  | Yes | 3,633 | 8.0 | 281 | 2.5 | 4,188 | 10.4 | 897 | 7.0 |
| Ever worked as trader | No | 44,820 | 98.2 | 11,148 | 99.7 | 39,209 | 97.7 | 12,780 | 99.4 |
|  | Yes | 841 | 1.8 | 33 | 0.3 | 933 | 2.3 | 77 | 0.6 |
| Ever worked as co-dependent or professionals | No | 44,425 | 97.3 | 10,893 | 97.4 | 38,254 | 95.3 | 12,443 | 96.8 |
|  | Yes | 1,236 | 2.7 | 288 | 2.6 | 1,888 | 4.7 | 415 | 3.2 |
| Cumulative duration of periods of non-employment | < 12 months | 20,086 | 44.0 | 8,399 | 75.1 | 19,299 | 48.1 | 9,527 | 74.1 |
|  | 1-4 years | 16,145 | 35.4 | 2,365 | 21.2 | 13,844 | 34.5 | 3,003 | 23.4 |
|  | 5-9 years | 7,336 | 16.1 | 381 | 3.4 | 5,593 | 13.9 | 298 | 2.3 |
|  | 10-14 years | 2,081 | 4.6 | 36 | 0.3 | 1,399 | 3.5 | 30 | 0.2 |
|  | ≥ 15 years | 13 | 0.0 | - | 0.0 | 6 | 0.0 | - | 0.0 |
| Prevailing work geographic area | Northwest | 11,995 | 26.3 | 4,366 | 39.0 | 9,931 | 24.7 | 4,884 | 38.0 |
|  | Northest | 8,798 | 19.3 | 3,034 | 27.1 | 7,419 | 18.5 | 3,103 | 24.1 |
|  | Central | 8,349 | 18.3 | 2,871 | 25.7 | 7,066 | 17.6 | 3,590 | 27.9 |
|  | South and Islands | 16,510 | 36.2 | 909 | 8.1 | 15,719 | 39.2 | 1,278 | 9.9 |
|  | Abroad | 9 | 0.0 | 0 | 0.0 | 7 | 0.0 | 1 | 0.0 |
| Prevailing Firm size | Missing | 66 | 0.1 | 35 | 0.3 | 23 | 0.1 | 18 | 0.1 |
|  | 1-9 | 18,778 | 41.1 | 6,295 | 56.3 | 17,148 | 42.7 | 6,967 | 54.2 |
|  | 10-19 | 9,032 | 19.8 | 1,875 | 16.8 | 7,935 | 19.8 | 2,327 | 18.1 |
|  | 20 - 199 | 13,549 | 29.7 | 2,434 | 21.8 | 11,049 | 27.5 | 3,007 | 23.4 |
|  | > 199 | 4,236 | 9.3 | 542 | 4.9 | 3,987 | 9.9 | 539 | 4.2 |

a: HIC - high income country b: SMPC - strong migratory pressure country

**Table 6 – Health status of workers in the construction industry.**

|  | | **2005** | | | | **2010** | | | |
| --- | --- | --- | --- | --- | --- | --- | --- | --- | --- |
|  |  | HIC^a^ | | SMPC^b^ | | HIC^a^ | | SMPC^b^ | |
|  |  | Person years | % | Person years | % | Person years | % | Person years | % |
| **Total** | | 45,661 | 100 | 11,182 | 100 | 40,142 | 100 | 12,857 | 100 |
|  | | | | | | | | | |
| Number of serious work injuries in the 5 years preceding the beginning of follow-up | 0 | 44,363 | 97.2 | 10,960 | 98.0 | 39,195 | 97.6 | 12,594 | 98.0 |
|  | 1 | 1,267 | 2.8 | 219 | 2.0 | 928 | 2.3 | 258 | 2.0 |
|  | 2 | 31 | 0.1 | 2 | 0.0 | 18 | 0.0 | 6 | 0.0 |
|  | 3+ | - | 0.0 | - | 0.0 | 1 | 0.0 | - | 0.0 |
| Number of hospital discharges in the 3 years preceding the beginning of follow-up | 0 | 36,794 | 80.6 | 10,133 | 90.6 | 32,998 | 82.2 | 11,767 | 91.5 |
|  | 1 | 6,386 | 14.0 | 837 | 7.5 | 5,317 | 13.2 | 852 | 6.6 |
|  | 2 | 1,630 | 3.6 | 158 | 1.4 | 1,238 | 3.1 | 174 | 1.4 |
|  | 3+ | 850 | 1.9 | 54 | 0.5 | 589 | 1.5 | 65 | 0.5 |

a: HIC - high income country b: SMPC - strong migratory pressure country
