# Supplementary material for: Differences in work injury risk between immigrants and natives: changes since the economic recession in Italy
Source: BMC Public Health. 2019 Jun 27;19:836. doi: 10.1186/s12889-019-7178-2 (PMC6598376; doi:10.1186/s12889-019-7178-2)
Supplement: Supplementary file 2 — Appropriateness of the matched samples (DOCX 36 kb) [file 12889_2019_7178_MOESM2_ESM.docx]

**Additional file 2**

**Appropriateness of the matched samples**

*Methods*

To evaluate the appropriateness of the matched samples and judge the success of propensity score modelling, standardized differences were calculated^^[[1]](#footnote-1)^^.

For each continuous covariate the degree of imbalance was calculated as follows:

$$d=100*\frac{\left|X_{SMPC}-X_{HIC}\right|}{\sqrt{\frac{S_{SMPC}^{2}+S_{HIC}^{2}}{2}}}$$

For dichotomous variables the standardized difference was defined as:

$$d=100*\frac{\left|P_{SMPC}-P_{HIC}\right|}{\sqrt{\frac{P_{SMPC}(1-P_{SMPC})+P_{HIC}(1-P_{HIC})}{2}}}$$

In addition to assess the balance of covariates a test proposed by Sianesi^^[[2]](#footnote-2)^^ was used. She suggested to re-estimate the propensity score on the matched sample and compare the pseudo-R2’s before and after the matching. The pseudo-R2 indicates how well the regressors explain the probability of being in the intervention group. After matching there should be no systematic differences in the distribution of covariates between both groups and therefore, the pseudo-R2 should be fairly low.

*Results*

The standardized differences were strongly reduced after the matching (table 1-3). Furthermore, the Sianesi-test confirmed that, after matching, there were no systematic differences in the distribution of covariates between groups. The pseudo-R2 calculated on the matched sample is close to zero.

**Table 1 - Standardized differences before and after the PS matching between HIC workers in 2005 and SMPC workers in 2005.**

|  | | **Metal working sector** | | **Construction sector** | |
| --- | --- | --- | --- | --- | --- |
|  |  | Std Differences before matching | Std Differences after matching | Std Differences before matching | Std Differences after matching |
| **Personal characteristics** | | | | | |
| Age | < 25 | - | - | - | - |
|  | 25-34 | 7.5 | 4.1 | 26.2 | 4.8 |
|  | 35-44 | 13.0 | -2.1 | 2.4 | 4.6 |
|  | 45-55 | -19.0 | -7.8 | -29.0 | -15.5 |
| **Data on the employment relationship** | | | | | |
| Skill level | Blue collar | - | - | - | - |
|  | Apprentice | -7.3 | 2.9 | -14.9 | 3.7 |
| Firm size (yearly average number of employees) | | -31.2 | -7.0 | -16.4 | -4.6 |
| Firm geographic area | Northwest | - | - | - | - |
|  | Northest | 27.7 | 0.5 | 20.2 | -0.8 |
|  | Central | -3.0 | -5.6 | 14.3 | -8.1 |
|  | South and Islands | -47.3 | -6.5 | -78.7 | -2.1 |
| Month in which the individual has entered the follow-up | January | - | - | - | - |
|  | February | 8.1 | -14.6 | 4.8 | -5.0 |
|  | March | 9.4 | -1.2 | 5.2 | -7.2 |
|  | April | 5.9 | 0.9 | 3.6 | -2.7 |
|  | May | 5.5 | 0.3 | 4.4 | 2.7 |
|  | June | 5.8 | -1.3 | 3.2 | -3.3 |
|  | July | 4.0 | -1.4 | 1.4 | 1.6 |
|  | August | 2.7 | -0.5 | 0.9 | 2.4 |
|  | September | 8.0 | -0.4 | 5.5 | -3.8 |
|  | October | 10.3 | -7.9 | 6.4 | 0.3 |
|  | November | 6.9 | -7.1 | 2.8 | -2.8 |
|  | December | 5.9 | 2.2 | 4.0 | -9.9 |
| Job tenure | | -76.6 | 0.1 | -43.9 | 5.9 |
| **Data on the working career (in the 20 years preceding the year)** | | | | | |
| Prevailing skill level | Apprentice | -11.6 | 3.9 | -25.3 | 3.5 |
|  | Blue-collar | - | - | - | - |
|  | White-collar | -13.6 | -1.9 | -12.1 | -0.7 |
| Flags that indicates whether one has ever worked as | Artisan | -19.4 | -2.6 | -23.6 | -6.6 |
|  | Trader | -12.7 | -1.2 | -15.0 | -1.3 |
|  | Economic dependent self employed or professional | 6.4 | -3.5 | 1.0 | -2.7 |
| Cumulative duration of the periods as employee | | -58.0 | -6.1 | -77.4 | -0.1 |
| Cumulative duration of the periods of non-employment | | -29.7 | -3.3 | -82.6 | -7.9 |
| Prevailingt economic sector | Metal working | - | - | - | - |
|  | Construction | 9.4 | 1.5 | 6.0 | -2.8 |
|  | Wholesale and retail trade | -9.0 | 3.1 | -7.4 | 3.6 |
|  | Transport and storage | 8.9 | 0.5 | -1.4 | -2.5 |
|  | Financial and real estate activities | 25.8 | -3.0 | 11.7 | 3.4 |
|  | Hotels and restaurants | 7.5 | -6.1 | 3.9 | 1.7 |
|  | Other manufacture sectors | 3.1 | 6.3 | -5.7 | 0.5 |
|  | Instruction and health services | 0.7 | -3.5 | -7.6 | -1.0 |
|  | Missing | 5.4 | -7.0 | -1.6 | 2.8 |
| Prevalent firm size | | -8.3 | -2.7 | -4.2 | -0.4 |
| Prevailing firm geographic area | Northwest | - | - | - | - |
|  | Northest | 27.9 | -0.5 | 19.6 | -0.1 |
|  | Central | -1.1 | -4.1 | 17.9 | -9.5 |
|  | South and Islands | -47.0 | -15.2 | -79.2 | -3.3 |
| Quartile of wage in the 5 years preceding the beginning of follow-up | I | - | - | - | - |
|  | II | 20.6 | 6.6 | 23.0 | -0.7 |
|  | III | -21.3 | 5.8 | 5.9 | 9.4 |
|  | IV | -53.9 | -0.7 | -32.2 | 6.0 |
| **Data on Health** **in the 5 years preceding the beginning of follow-up** | | | | | |
| Proportion of weeks of sick absence and of paid weeks | | -3.3 | -7.0 | -13.4 | -6.0 |
| Number of serious work injuries | | 4.2 | 1.2 | -5.7 | 2.3 |
| Number of hospital discharges in the 3 years preceding the beginning of follow-up | | -16.7 | -4.5 | -27.8 | -9.2 |

**Table 2 - Standardized differences before and after the PS matching between SMPC workers in 2010 and SMPC workers in 2005.**

|  | | **Metal working sector** | | **Construction sector** | |
| --- | --- | --- | --- | --- | --- |
|  |  | Std Differences before matching | Std Differences after matching | Std Differences before matching | Std Differences after matching |
| **Personal characteristics** | | | | | |
| Age | < 25 | - | - | - | - |
|  | 25-34 | 13.9 | 0.6 | 7.0 | 1.4 |
|  | 35-44 | -2.8 | 0.2 | -4.4 | 0.6 |
|  | 45-55 | -18.3 | -0.7 | -7.0 | -0.6 |
| **Data on the employment relationship** | | | | | |
| Skill level | Blue collar | - | - | - | - |
|  | Apprentice | 2.2 | -0.3 | -5.4 | 1.0 |
| Firm size (yearly average number of employees) | | 4.2 | 0.3 | 2.6 | 0.4 |
| Firm geographic area | Northwest | - | - | - | - |
|  | Northest | 3.3 | 0.5 | 10.0 | 0.0 |
|  | Central | -1.5 | -0.3 | -8.4 | -0.9 |
|  | South and Islands | -7.6 | -0.4 | -11.2 | -0.1 |
| Month in which the individual has entered the follow-up | January | - | - | - | - |
|  | February | 6.3 | -0.7 | 7.1 | 0.4 |
|  | March | 7.2 | 0.2 | 1.7 | 0.1 |
|  | April | 4.0 | 0.0 | 3.2 | -0.8 |
|  | May | -0.2 | -0.1 | 5.8 | -0.1 |
|  | June | 2.5 | -0.6 | 1.4 | 0.2 |
|  | July | -0.9 | -0.3 | 0.4 | -0.5 |
|  | August | -3.6 | -0.1 | -1.7 | -0.1 |
|  | September | 3.3 | -0.4 | 0.3 | -0.3 |
|  | October | 2.5 | -0.2 | 2.3 | 0.0 |
|  | November | 3.9 | -1.0 | 3.7 | 0.3 |
|  | December | 1.1 | 0.1 | 5.0 | -0.9 |
| Job tenure | | -32.5 | -2.6 | -27.4 | -0.1 |
| **Data on the working career (in the 20 years preceding the year)** | | | | | |
| Prevailing skill level | Apprentice | 3.1 | 0.2 | -12.5 | 2.1 |
|  | Blue-collar | - | - | - | - |
|  | White-collar | -2.4 | 0.3 | 0.5 | 0.1 |
| Flags that indicates whether one has ever worked as | Artisan | -11.9 | -0.4 | -23.1 | 0.0 |
|  | Trader | -3.9 | 0.2 | -5.4 | -0.1 |
|  | Economic dependent self employed or professional | -2.2 | 1.6 | -3.3 | 1.8 |
| Cumulative duration of the periods as employee | | -12.2 | 0.0 | -30.9 | 0.1 |
| Cumulative duration of the periods of non-employment | | 3.9 | -1.4 | -0.8 | -1.9 |
| Prevailing economic sector | Metal working | - | - | - | - |
|  | Construction | -2.2 | 0.3 | -25.5 | 1.0 |
|  | Wholesale and retail trade | 2.0 | 0.0 | 7.4 | -0.1 |
|  | Transport and storage | 7.2 | -0.3 | 6.2 | -1.5 |
|  | Financial and real estate activities | 7.2 | -1.2 | 9.6 | -0.3 |
|  | Hotels and restaurants | 5.0 | 0.0 | 6.3 | -1.0 |
|  | Other manufacture sectors | 12.6 | -0.8 | 13.0 | 0.5 |
|  | Instruction and health services | 2.7 | -0.2 | 2.3 | -1.4 |
|  | Missing | 9.0 | -0.2 | 6.6 | 1.2 |
| Prevalent firm size | | 0.2 | 0.0 | 3.5 | -0.5 |
| Prevailing firm geographic area | Northwest | - | - | - | - |
|  | Northest | 0.6 | 0.3 | 7.1 | -1.5 |
|  | Central | 1.0 | 0.3 | -4.7 | 0.1 |
|  | South and Islands | -3.0 | 0.2 | -8.9 | 1.0 |
| Quartile of wage in the 5 years preceding the beginning of follow-up | I | - | - | - | - |
|  | II | 6.8 | -0.6 | 3.5 | 0.7 |
|  | III | -2.0 | -0.6 | 1.9 | 0.1 |
|  | IV | -4.9 | -2.9 | -2.8 | -1.0 |
| **Data on Health** **in the 5 years preceding the beginning of follow-up** | | | | | |
| Proportion of weeks of sick absence and of paid weeks | | 9.0 | -0.5 | 10.9 | 3.1 |
| Number of serious work injuries | | 0.7 | 0.3 | -0.1 | 0.2 |
| Number of hospital discharges in the 3 years preceding the beginning of follow-up | | 5.6 | -0.3 | 3.3 | 0.4 |

**Table 3 - Standardized differences before and after the PS matching between HIC workers in 2010 and SMPC workers in 2005.**

|  | | **Metal working sector** | | **Construction sector** | |
| --- | --- | --- | --- | --- | --- |
|  |  | Std Differences before matching | Std Differences after matching | Std Differences before matching | Std Differences after matching |
| **Personal characteristics** | | | | | |
| Age | < 25 | - | - | - | - |
|  | 25-34 | 20.8 | 10.3 | 32.5 | 12.3 |
|  | 35-44 | 2.4 | -2.0 | -2.9 | -5.2 |
|  | 45-55 | -32.1 | -8.2 | -41.9 | -1.0 |
| **Data on the employment relationship** | | | | | |
| Skill level | Blue collar | - | - | - | - |
|  | Apprentice | -1.7 | -3.2 | -1.4 | -7.7 |
| Firm size (yearly average number of employees) | | -29.6 | -5.6 | -11.8 | -0.8 |
| Firm geographic area | Northwest | - | - | - | - |
|  | Northest | 27.3 | -6.1 | 27.5 | 10.0 |
|  | Central | -3.8 | 1.1 | 15.9 | -19.9 |
|  | South and Islands | -50.9 | -4.0 | -93.3 | 0.0 |
| Month in which the individual has entered the follow-up | January | - | - | - | - |
|  | February | 11.6 | -8.1 | 6.7 | -0.8 |
|  | March | 11.9 | -5.1 | 4.9 | -1.9 |
|  | April | 8.4 | -7.8 | 6.9 | -4.2 |
|  | May | 8.1 | -6.5 | 7.1 | -1.7 |
|  | June | 8.6 | -4.1 | 5.4 | -15.0 |
|  | July | 6.3 | -0.5 | -0.4 | -2.2 |
|  | August | 5.0 | -2.8 | 0.9 | -0.4 |
|  | September | 9.1 | -7.3 | 5.4 | -7.1 |
|  | October | 10.7 | -12.7 | 6.8 | -16.0 |
|  | November | 7.4 | -9.7 | 2.9 | 0.8 |
|  | December | 7.2 | -4.7 | 1.7 | 3.5 |
| Job tenure | | -93.6 | 1.1 | -52.5 | 11.1 |
| **Data on the working career (in the 20 years preceding the year)** | | | | | |
| Prevailing skill level | Apprentice | -1.9 | 4.9 | -13.6 | 2.0 |
|  | Blue-collar | - | - | - | - |
|  | White-collar | -14.7 | -1.8 | -13.2 | -0.5 |
| Flags that indicates whether one has ever worked as | Artisan | -21.3 | -1.9 | -33.1 | -3.2 |
|  | Trader | -13.2 | -3.3 | -17.9 | -1.1 |
|  | Economic dependent self employed or professional | -5.1 | 1.4 | -9.4 | 6.7 |
| Cumulative duration of the periods as employee | | -66.0 | -9.6 | -89.7 | -5.1 |
| Cumulative duration of the periods of non-employment | | -18.9 | -4.4 | -76.1 | -2.5 |
| Prevailing economic sector | Metal working | - | - | - | - |
|  | Construction | 9.4 | 4.3 | -7.3 | 2.6 |
|  | Wholesale and retail trade | -5.5 | 0.5 | -3.2 | 3.6 |
|  | Transport and storage | 9.3 | 6.5 | 1.5 | 3.9 |
|  | Financial and real estate activities | 25.6 | -7.6 | 12.7 | 6.4 |
|  | Hotels and restaurants | 10.1 | -9.3 | 4.4 | -7.6 |
|  | Other manufacture sectors | 9.3 | 0.3 | 1.5 | -9.8 |
|  | Instruction and health services | 0.9 | 2.0 | -4.4 | -2.1 |
|  | Missing | 8.2 | -10.8 | 3.4 | 4.4 |
| Prevalent firm size | | -10.6 | -2.4 | -4.1 | -4.2 |
| Prevailing firm geographic area | Northwest | - | - | - | - |
|  | Northest | 25.7 | -6.1 | 24.7 | 5.9 |
|  | Central | -0.1 | 2.2 | 20.5 | -19.4 |
|  | South and Islands | -49.1 | -8.7 | -92.9 | -7.9 |
| Quartile of wage in the 5 years preceding the beginning of follow-up | I | - | - | - | - |
|  | II | 20.4 | 10.3 | 20.2 | -4.8 |
|  | III | -22.2 | 7.8 | 5.6 | 5.0 |
|  | IV | -57.1 | 0.6 | -31.6 | 3.8 |
| **Data on Health** **in the 5 years preceding the beginning of follow-up** | | | | | |
| Proportion of weeks of sick absence and of paid weeks | | -0.7 | -6.3 | -8.5 | -0.6 |
| Number of serious work injuries | | 7.0 | -5.7 | -2.1 | -1.0 |
| Number of hospital discharges in the 3 years preceding the beginning of follow-up | | -14.0 | -0.6 | -23.1 | 2.2 |

1. Heinze G, Juni P. An overview of the objectives of and he approaches to propensity score analyses. Eur Heart J 2011:32(14):1704-1708. doi: 10.1093/eurheartj/ehr031. [↑](#footnote-ref-1)
2. Sianesi, B. An evaluation of the Swedish system of active labor market programs in the 1990s. The Review of Economics and Statistics 2004, 86(1), 133-155. [↑](#footnote-ref-2)
